# Activity of *Palythoa caribaeorum* Venom on Voltage-Gated Ion Channels in Mammalian Superior Cervical Ganglion Neurons

**DOI:** 10.3390/toxins8050135

**Published:** 2016-05-05

**Authors:** Fernando Lazcano-Pérez, Héctor Castro, Isabel Arenas, David E. García, Ricardo González-Muñoz, Roberto Arreguín-Espinosa

**Affiliations:** 1 Departamento de Química de Biomacromoléculas, Instituto de Química, Universidad Nacional Autónoma de México, Av. Universidad 3000, Ciudad Universitaria, Ciudad de México 04510, Mexico; 2 Departamento de Fisiología, Facultad de Medicina, Universidad Nacional Autónoma de México, Av. Universidad 3000, Ciudad Universitaria, Ciudad de México 04510, Mexico; hectorcastrompss@hotmail.com (H.C.); isabel.arenas@unam.mx (I.A.); erasmo@unam.mx (D.E.G.); 3 Laboratorio de Biología de Cnidarios, Facultad de Ciencias Exactas y Naturales, Universidad Nacional de Mar del Plata, Funes 3250, Mar del Plata 7600, Argentina; ricordea.gonzalez@gmail.com

**Keywords:** cnidaria, zoanthids, *Palythoa caribaeorum*, voltage-gated ion channels, SCG neurons, MALDI-TOF-MS

## Abstract

The Zoanthids are an order of cnidarians whose venoms and toxins have been poorly studied. *Palythoa caribaeorum* is a zoanthid commonly found around the Mexican coastline. In this study, we tested the activity of *P. caribaeorum* venom on voltage-gated sodium channel (Na_V_1.7), voltage-gated calcium channel (Ca_V_2.2), the A-type transient outward (I_A_) and delayed rectifier (I_DR_) currents of K_V_ channels of the superior cervical ganglion (SCG) neurons of the rat. These results showed that the venom reversibly delays the inactivation process of voltage-gated sodium channels and inhibits voltage-gated calcium and potassium channels in this mammalian model. The compounds responsible for these effects seem to be low molecular weight peptides. Together, these results provide evidence for the potential use of zoanthids as a novel source of cnidarian toxins active on voltage-gated ion channels.

## 1. Introduction

Cnidarians comprise approximately 10,000 species within five different classes: Anthozoa (sea anemones, corals, zoanthids), Scyphozoa (jellyfishes), Cubozoa (box jellyfishes), Hydrozoa and Staurozoa [1]. These organisms are mainly carnivorous although some species can use photosynthesis as an alternative feeding source facilitated by symbiont zooxantelles living within their tissues [2]. All cnidarians are considered to be toxic as they possess specialized organelles called nematocysts where they store a venom which is known to contain a complex mixture of compounds including bioactive amines, steroids, glycoproteins and cytolitic proteins [3], together with an extensive variety of neurotoxic peptides acting on voltage-gated channels [4,5].

Cnidarian venoms need to contain neurotoxins in order to paralyze their prey by directly affecting ion channels and receptors, neuromuscular junctions and nerve cell membranes [6]. The best-known cnidarian neurotoxins are the low molecular weight peptides isolated from sea anemones [7]. These compounds can affect a variety of targets such as voltage-gated sodium and potassium channels, Acid Sensing Ion Channel 3 (ASIC3) and Transient Receptor Potential Vanilloid 1 (TRPV1) [8].

There is currently little known about venoms of anthozoan species other than sea anemones. The zoanthids comprise a group of cnidarians characterized by colonies of clonal polyps possessing two rows of tentacles. They are usually found in shallow zones of coral reefs [9]. Their venoms have received little attention although they may be of greater importance. However, extracts and compounds isolated from them exhibit several biological activities [10,11]. Recently, a low molecular weight fraction of the zoanthid *Zoanthus* *sociatus* has been shown to be lethal to mice at a concentration of 792 µg/kg and to accelerate KCl-induced lethality [12]. Furthermore, the same fraction was found to be a potential negative modulator of voltage-gated Ca^2+^ channels inhibiting the release of insulin in pancreatic β-cells [13]. Likewise, a 3.4 kDa peptide isolated from the zoanthid *Palythoa caribaeorum* caused a delay on the sodium current inactivation in voltage-gated sodium channels Na_v_1.7 of the superior cervical ganglion neurons of the rat [14]. To our knowledge, no other characterization of zoanthids venoms on voltage-gated ion channels has been carried out.

**Figure 1:**
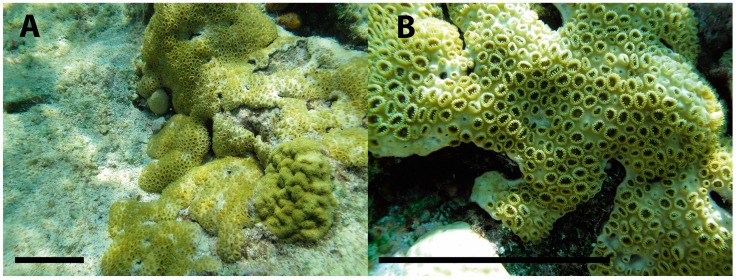
The zoanthid *Palythoa caribaeorum*: (**A**) *P. caribaeorum* in its natural habitat; (**B**) Open *P.* *caribaeorum* polyps showing tentacles. Scale bar: 10 cm.

Therefore, we assessed the biological activity of *P. caribaeorum* venom (Figure 1) on voltage-gated ion channels (Na_V_1.7, Ca_V_2.2 and on I_A_ and I_DR_ K^+^ currents) by means of electrophysiological techniques. Moreover, the molecular weight distribution of the venom peptides was observed by Matrix Assisted Laser Desorption Ionization Time-of-Flight (MALDI-TOF-MS) spectrometry. Together, these results show the presence of low molecular weight peptides with activity on voltage-gated ion channels which suggests that zoanthids are useful in searching for novel biomolecules.

## 2. Results and Discussion

**Figure 2:**
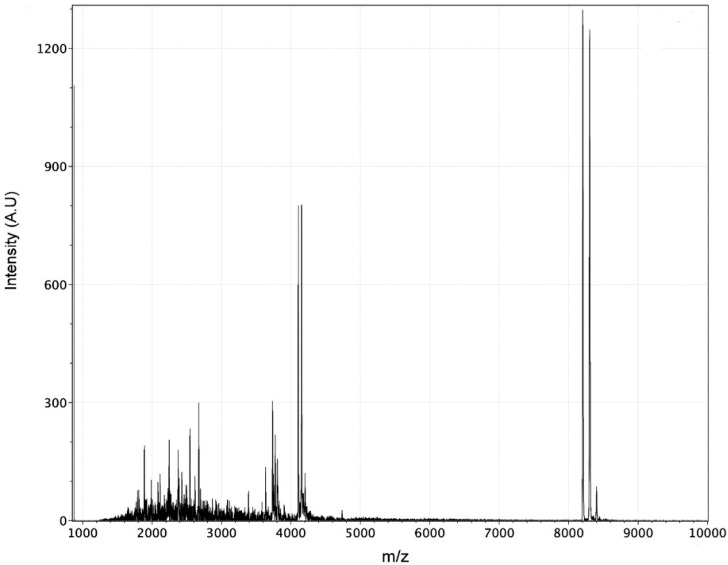
Matrix-assisted laser-desorption/ionization time-of-flight mass spectrometry (MALDI-TOF) of *P. caribaeorum* venom.

Since cnidarians are carnivorous animals, they need to capture their prey and immobilize them. For this reason, their venoms contain a mixture of toxins that disrupt the function of the nervous system of the prey, for example fish and crustaceans. It is well known that anthozoan neurotoxins, especially those isolated from sea anemones, modulate ion channels on excitable cells. Nevertheless, the activity of zoanthid venoms remains unknown. The MALDI-TOF mass spectrometry analysis showed that the venom contains low molecular weight peptides (within a mass range of 1800 to 9000 Da) (Figure 2).

### 2.1. Effect of P. caribaeorum Venom on Na_V_1.7 Sodium Channel Current

**Figure 3:**
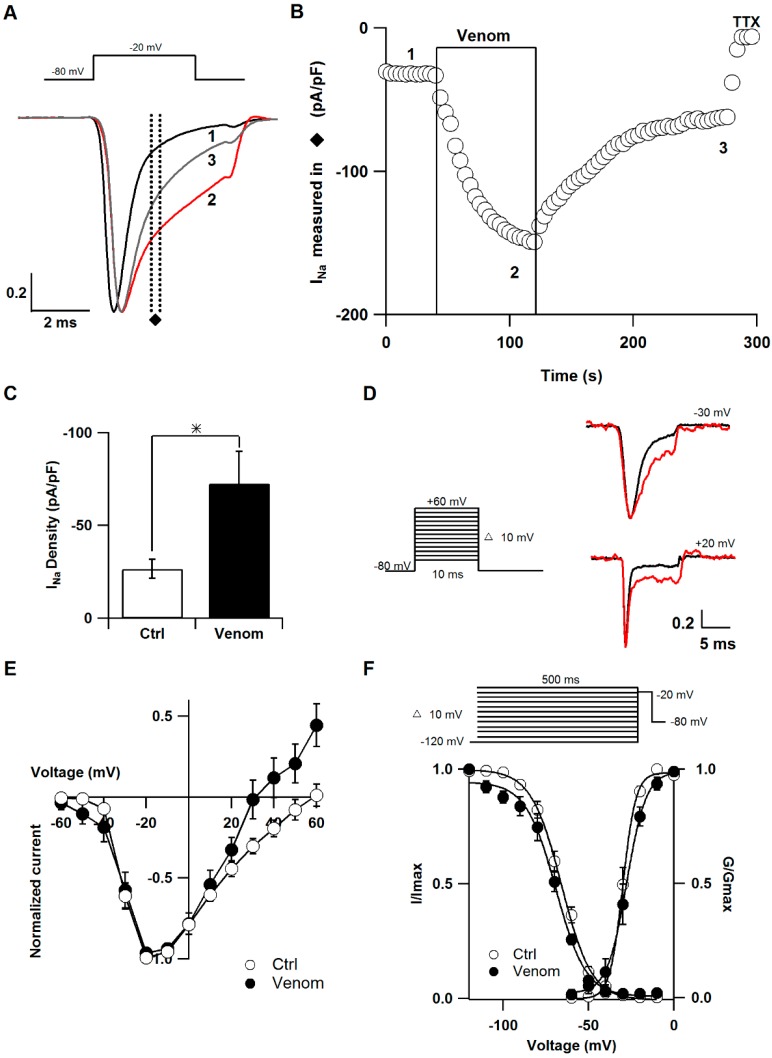
Effect of *P. caribaeorum* venom on sodium current: (**A**) Normalized representative sodium current traces before (1), during (2) and after (3) application of venom. Current amplitude was measured between the dotted lines (♦); (**B**) Time course of sodium current amplitude. The application of venom is indicated within a box. Current changes were compared to the total block observed in the presence of tetrodotoxin (TTX); (**C**) Mean current density after venom addition and under control conditions (Ctrl); (**D**) Voltage protocol used for measuring I-V relations and normalized representative current traces at −30 mV and +20 mV before (black) and during (red) venom exposure; (**E**) I-V relationship was elicited by voltage steps between −60 and +60 mV in 10 mV increments before and during the venom application; (**F**) Steady-state inactivation and activation curves under control conditions and after venom application (○ Ctrl and ● Venom). Data points for both activation and inactivation were well fitted to the single Boltzmann equation. * represents *p* < 0.05.

Voltage-gated sodium channels (Na_V_) were the first target discovered for cnidarian neurotoxins [15] and more than 60 sea anemone toxins affecting this channel have been reported to date [8]. Thus, the effect of the venom on Na_V_1.7 channels in SCG neurons of the rat was examined in this study [16,17,18]. Sodium currents were elicited with a square pulse from −80 to −20 mV every four seconds. Figure 3A shows the superimposed sodium current traces before, during and after application of the venom. The current amplitude was measured as the mean data points between 1.7 and 2 ms after the pulse onset. The venom increased the amplitude during the decay phase and the effect was partially reversed after washout (Figure 3B). As current amplitude depends on cell size, we compared the density of the current dividing the amplitude by the cell capacitance. Current density was significantly larger after venom application compared to control conditions (venom 72.7 ± 17.2 pA/pF; control 26.6 ± 5.06 pA/pF) (*n* = 9) (Figure 3C).

Sea anemone neurotoxins are the best characterized in terms of mode of action and kinetics [19]. Thus, we also evaluated the effect of the venom on activation and inactivation of the sodium channel (Figure 3D–F). Steady-state inactivation of the sodium current was measured using a double pulse protocol in which a 500 ms conditioning pulse preceded a 5 ms test pulse to −20 mV [20]. The midpoint (*V_h_*) and slope factor (*k*) were obtained using a Boltzmann function of individual cells. Under control conditions, the *V_h_* average was −66.05 ± 1.5 mV, whereas it was −69.8 ± 1.7 mV after venom application (*n* = 5). Moreover, *k* was 8.7 ± 0.5 mV and 8.9 ± 1.2 mV for control and venom conditions, respectively (*n* = 5). Therefore, venom treatment did not significantly shift steady-state inactivation. For the activation curve, conductance was calculated from current-voltage (I-V) relationship using the equation G_Na_ = I_Na_/(V − V_Na_), where I_Na_ is the peak amplitude of current, V is the membrane potential and V_Na_ is the reversal potential [21]. A Boltzmann equation was used to obtain *V_h_* and *k*, as follows:

(1) $$\frac{G}{G\text{max}}\;={\left\{1+\text{exp}\left[\frac{Vh-V}{k}\right]\right\}}^{-1}$$

where *G*_max_ is the maximal conductance. Significant differences were not observed at mean values of *V_h_* (−28.1 ± 1.8 and −29.6 ± 1.05 mV for venom and control conditions, respectively) (*n* = 6). Conversely, when venom was applied, *k* increased significantly compared to control. (Venom 5.2 ± 0.2 and 3.5 ± 0.2 mV for control conditions) (*n* = 6).

These results suggest that *P. caribaeorum* venom contains at least one neurotoxin that slows the inactivation kinetic of the sodium current in SCG neurons. This effect is consistent with the slow inactivation induced by all sea anemone toxins known to date. This finding allows us to think that sodium channel neurotoxins from other cnidarian groups might work the same way [16,22]. In addition, an increase in the slope of the activation curve and a slight shift to the right of peak sodium current were observed. It is likely that the venom has an effect on the movement of the voltage-sensor, modifying not only the inactivation but also the activation process. However, it is not possible to discern whether a different peptide is responsible for this effect.

### 2.2. Effect of P. caribaeorum Venom on I_A_ and I_DR_ Potassium Currents

**Figure 4:**
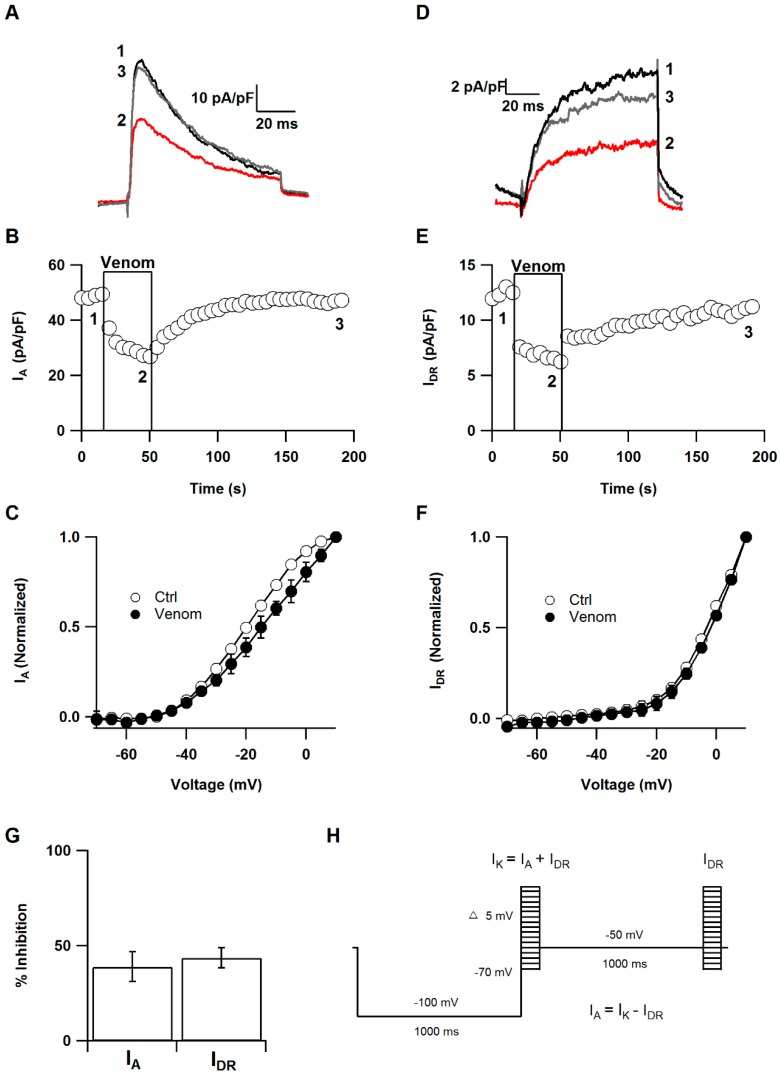
I_A_ and I_DR_ are inhibited by *P. caribaeorum* venom: (**A**,**D**) Representative I_A_ and I_DR_ traces before (1), during (2) and after (3) venom addition; (**B**,**E**) Time courses of the venom effect on the amplitude of potassium currents in SCG’s neurons. Exposure to venom is marked with a box; (**C**,**F**) Effect of venom superfusion on I-V relationship of I_A_ and I_DR_; (**G**) Aggregated data for inhibition of potassium currents (*n* = 5); (**H**) I_A_ and I_DR_ K^+^ currents voltage protocol.

A major target for sea anemone toxins are voltage-gated potassium channels [8], therefore, the effect of *P. caribaeorum* extract was also tested on them. SCG neurons display several outward K^+^ current components: a transient current (I_A_), a Ca^2+^ dependent component and a sustained K^+^ current that is the delayed rectifier (I_DR_) [23]. These currents play a key role in the control of excitability, synaptic input, and neurotransmitter release [24,25]. Therefore, the effect of *P. caribaeorum* venom on I_A_ and I_DR_ was evaluated. I_A_ and I_DR_ were isolated using a voltage pulse protocol every five seconds. This protocol measured the total potassium current (I_A_ + I_DR_) by a 80 ms pulse to −20 mV after a 1 s conditioning prepulse to −100 mV. Subsequently, the I_A_ was isolated from the total potassium current by a depolarizing 1 s conditioning prepulse to −50 mV. Therefore, the remaining outward current evoked by 80 ms pulse to −20 mV is mostly comprised of I_DR_. Finally, the I_A_ was calculated by digital subtraction of I_DR_ from total potassium current (Figure 4H) [23]. The current amplitude of I_A_ was measured from the peak, and amplitude of I_DR_ was determined as the mean current value of data points during the last 5 ms test pulse. The venom inhibited 38.8% ± 7.8% the current amplitude of I_A_ without altering I-V relationship (Figure 4A–C,G) (*n* = 5). Likewise, I_DR_ was inhibited 43.7% ± 5.3% and the voltage dependence was not shifted (Figure 4D–G) (*n* = 5).

These results indicate that *P. caribaeorum* venom affects these two different types of potassium currents in a voltage-independent manner. In SCG neurons, I_A_ is produced by K_V_1.4, K_V_4.1, K_V_4.2 and K_V_4.3 channels [26], while I_DR_ is associated with Kv2 family [27]. Quite a few cnidarian toxins have been tested on K_V_ channels associated with this current. BDS-I and II isolated from the sea anemone *Anemonia sulcata* were first reported as specific blockers of the fast inactivating potassium channel K_V_3.4 [28]. However, their specificity was later questioned and it was shown that they inhibit K_V_3.1 and K_V_ 3.2 channels, which are ascribed to the delayed rectifier current [29]. ShK and APETx1, from *Stichodactyla helianthus* and *Anthopleura elegantissima*, respectively, are potent delayed rectifier K_V_ channels inhibitors, however, they have also been shown to inhibit Kv1.4 channels [30,31]. Most sea anemone potassium blockers described to date have been tested on I_DR_ related potassium channels [30].

### 2.3. Effect of P. caribaeorum Venom on Ca_V_2.2 Calcium Channel Current

**Figure 5:**
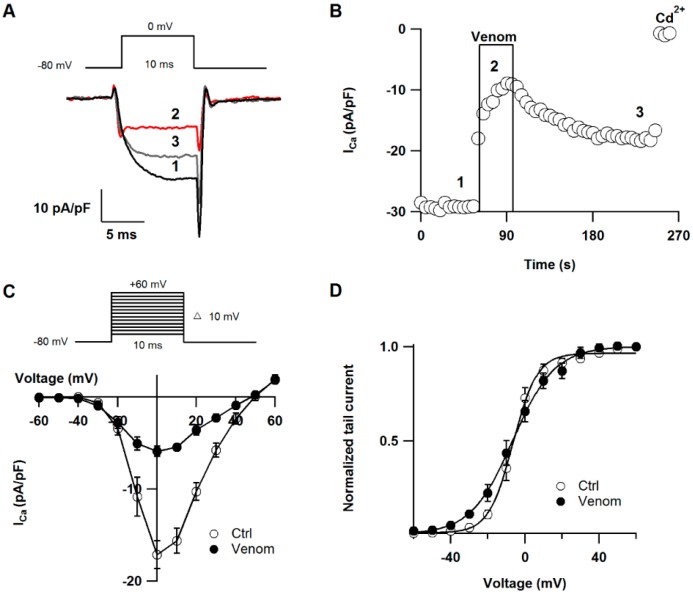
Cav 2.2 calcium current is inhibited by venom of *P. caribaeorum*: (**A**) Superimposed current traces normalized by the cell capacitance before (1), during (2) and after (3) venom application; (**B**) Time course of the venom effect on calcium current amplitude. The box indicates the time of the venom application; (**C**) I-V relationship was obtained by depolarizing pulses between −60 mV and +60 mV in 10 mV increments; (**D**) Activation curve measured at the peak of the tail current and normalized. Solid line is the best Boltzmann fit to data points.

The venom was also tested on calcium channels with the rationale that sodium and calcium channels share some structure features and this fraction may have activity on both type of channels [32]. SGC neurons contain mainly Ca_V_2.2 calcium channels and a small fraction of Ca_V_1 (L-type) channels. To eliminate the L-type current component, 5 µM nifedipine was added to the bath solution [33]. Ca_V_2.2 current was elicited with a voltage step from −80 to 0 mV every four seconds and was defined as the component of the current sensitive to 100 µM CdCl_2_. Figure 5A shows representative calcium current traces before, during and after venom application. Steady-state current amplitude was calculated as the mean value of data points between 5 and 6 ms after the onset of the test pulse. Superfusion of the venom inhibited 65.19% ± 3.52% the calcium current in less than 40 seconds and the effect was partially reversed after washout (*n* = 5) (Figure 5B). We also examined the effect of *P.* *caribaeorum* venom on I-V relationship and activation curve (Figure 5C,D). The extract inhibited current over the wide range of potentials and it showed a significant effect on slope factor of activation curve (5.2 ± 0.7 and 11.1 ± 1.4 mV, under control and venom conditions, respectively) (*n* = 7). However, the venom did not significantly affect the midpoint of activation (venom *V_h_* = −6.06 ± 2.4 mV, and −7.8 ± 1.5 mV in control conditions) (*n* = 7). Although the venom of *P. caribaeorum* did not affect significantly the midpoint of activation of Ca_V_ 2.2 calcium channel current, it readily inhibited this channel current influx suggesting an action affecting either the channel pore, *i.e.*, the surface charge, or the voltage-sensor machinery. According to this inhibition and the slope change, these data are in good agreement with a mechanism involving a trapping of the voltage-sensor of the channel. Toxin effects on movement of the voltage-sensor have been reported in calcium channels, e.g., ω-Aga-IVA decreases steepness of P-type calcium channel activation curve [34]. In fact, the voltage-sensor trapping has been proposed as a common mechanism of action of all toxins that alter the voltage-dependence of ion channel gating [22].

Despite almost all venomous animals produce calcium channel blockers, none of these blockers have been isolated from cnidarians [35]. These results clearly showed that the venom of *P. caribaeorum* inhibits Ca_V_2.2 channels, thus they reveal the first cnidarian venom known inhibiting calcium channels. Procedures are being designed to isolate the molecule eliciting this response.

## 3. Conclusions

Marine venoms are a rich source of voltage-gated channels modulators. Among these, research on sea anemone venoms has been largely investigated on Nav and Kv channel toxins, which have been shown to be promising tools on pharmacological research. Our results have shown that *P. caribaeorum* contains voltage-gated ion channel toxins and this opens the possibility to consider zoanthids as a new source of biologically active molecules. Moreover, this study evidences that cnidarians can also produce voltage-gated calcium channel toxins as other groups of animals like spiders and cone snails. Further studies may help in the advance of the knowledge of the action of cnidarian venoms acting on voltage-gated calcium channels.

## 4. Experimental Section

### 4.1. Venom Extraction

*Palythoa caribaeorum* organisms were collected by free diving in La Gallega coral reef in Veracruz, México. They were carefully separated from the rock using a chisel and a hammer, avoiding inducing mechanical stress in the polyps. In the laboratory, remaining rock was cleaned from the material, which was then soaked in water to eliminate superficial mucus. In order to extract nematocyst venom, the organisms were carefully squeezed in deionized water to expose hidden polyp tentacles, which were mechanically discharged. The solution was then centrifuged twice at 70,000 *g* for 15 min at 4 °C. The supernatant was lyophilized, and stored at −70 °C prior to use.

### 4.2. Mass Spectrometry Analysis

The venom was analyzed using MALDI-TOF. 5 µL of a saturated solution of sinapinic acid (>99.0% purity for MALDI-MS, Sigma, St. Louis, MO, USA) were added to 5 µg of lyophilized venom. 1 µL of this solution was deposited onto the MALDI plate and allowed to dry at room temperature. The spectrum was recorded on linear positive mode on a mass spectrometer (Microflex Bruker Daltonics, Bremen, Germany) equipped with nitrogen laser λ = 337 nm and a 20 kV acceleration voltage.

### 4.3. Superior Cervical Ganglion Neuron Culture

Superior cervical ganglion (SCG) neurons were isolated from 5 week-old male Wistar rats. Animals were obtained from the animal breeding facility of the School of Medicine at the Universidad Nacional Autónoma de México (UNAM) and were handled according to the Mexican Official Norm for Use, Care and Reproduction of Laboratory Animals NOM-062-ZOO-1999. The Research and Ethics Committees of School of Medicine, UNAM, approved the project´s experimental protocol, identification code: 067/2012, at 21 August 2012. Rats were anaesthetized with CO_2_ and decapitated with a guillotine. After dissection, ganglia were sliced into eight pieces. The tissue was incubated in Hank’s Balanced Salt Solution (HBSS) supplemented with 20 U/mL papain for 20 min at 37 °C. The sample was then transferred into a solution containing 1 mg/mL collagenase type I and 10 mg/mL dispase. This was then incubated for 20 min before mechanically disaggregating the tissue. The cell suspension was centrifuged at 180 *g* for 3 min and washed twice in Leibovitz’s L-15 medium and once in Dulbecco’s modified Eagle’s medium (DMEM), both supplemented with 10% (*v*/*v*) heat-inactivated fetal bovine serum and 1% penicillin-streptomycin solution. Cells were then plated on polystyrene culture dishes coated with poly-l-lysine and incubated in a humidified atmosphere of 95% air and 5% CO_2_ at 37 °C. Cells were used up to 24 h after plating. L-15 and DMEM culture media were obtained from Invitrogen Corp. (Carlsbad, CA, USA), and all other reagents were obtained from Sigma (St. Louis, MO, USA).

### 4.4. Electrophysiology Recording and Data Analysis

Calcium, sodium, and potassium currents were recorded in the whole cell configuration of the patch-clamp technique [21] with an EPC9 amplifier (HEKA Instruments Inc., Holliston, MA, USA) at room temperature (22–25 °C). Borosilicate glass pipettes (Kimble Chase, Vineland, NJ, USA) with a resistance of 1.6–2.2 MΩ were used. Series resistance was 3–7 MΩ and compensated to >60%. Currents were sampled at 100 kHz. Current recordings were filtered at 2.9 kHz. Cells were continuously bathed with control or test solutions at a rate of 2 mL/min. For measuring Na^+^ currents, the bath solution contained (in mM): 103 TEA-Cl, 60 NaCl, 10 HEPES, 8 glucose, 2.9 MgCl_2_, and 0.1 CdCl_2_, pH adjusted to 7.4 with TEA-OH. Pipette solution contained (in mM): 22 TEA-Cl, 140 CsCl, 10 HEPES, 0.1 BAPTA tetracesium, 1 MgCl_2_, 10 NaCl, 4 Mg_2_ATP, 0.3 GTP, and 0.1 leupeptin, pH adjusted to 7.4 with CsOH. For Ca^2+^ currents, the bath solution contained (in mM): 160 NaCl, 2.5 KCl, 10 HEPES, 8 Glucose, 5 CaCl_2_, 1 MgCl_2_, 0.005 nifedipine and 100 nM TTX, pH adjusted to 7.4 with NaOH. Pipette solution contained (in mM): 140 CsCl, 20 TEA-Cl, 10 HEPES, 0.1 BAPTA tetracesium, 1 MgCl_2_, 4 Mg_2_ATP, 3 GTP, and 0.1 leupeptin, pH adjusted to 7.4 with KOH. For K^+^ currents, the bath solution contained (in mM): 163 NaCl, 5 KCl, 2.9 MgCl_2_, 10 HEPES, 8 Glucose, 0.1 CdCl_2_, and 100 nM TTX, pH adjusted to 7.4 with NaOH. Pipette solution contained (in mM): 175 KCl, 5 MgCl_2_, 5 HEPES, 0.1 BAPTA tetrapotassium, 4 Mg_2_ATP, 0.3 GTP, and 0.1 leupeptin, pH adjusted to 7.4 with KOH. Na^+^ and Ca^2+^ currents were recorded in the presence of 100 nM TTX, and 0.1 mM CdCl_2_ as positive controls, respectively. The *P. caribaeorum* venom was diluted with bath solution to a final concentration of 1.25 mg/mL.

Data were analyzed using IGOR Pro software (Version 6.1.2.1, WaveMetrics, Portland, OR, USA, 2009). Results were expressed as mean ± standard error mean (SEM) and analyzed using Student’s *t* tests. Statistical significance was taken to *p* < 0.05.
